# Stagnation in Decreasing Gastric Cancer Incidence and Mortality in Quito: Time Trend Analysis, 1985–2013

**DOI:** 10.1155/2019/1504894

**Published:** 2019-02-26

**Authors:** Wilmer Tarupi, Esther de Vries, Patricia Cueva, José Yépez

**Affiliations:** ^1^Facultad de Ciencias de la Salud Eugenio Espejo, Universidad UTE, Quito, Ecuador; ^2^Department of Clinical Epidemiology and Biostatistics, Pontificia Universidad Javeriana, Bogotá, Colombia; ^3^Registro Nacional de Tumores, Sociedad de Lucha contra el Cáncer (SOLCA), Quito, Ecuador

## Abstract

**Background:**

Despite the significant global decline in mortality and incidence, gastric cancer (GC) remains a very common cause of illness and death in the Latin American region. This article seeks to describe, in depth, the time trend of incidence and mortality of GC in the city of Quito, from 1985 to 2013.

**Methods:**

Using data from the Quito Cancer Registry, annual sex-specific age-standardized incidence and mortality rates were calculated. The analysis included all types of GC together, as well as by histological subtype. Joinpoint regression analysis was performed to estimate the annual percentage change (EAPC). To evaluate cohort and period effects, Age-Period-Cohort (APC) modeling was performed.

**Results:**

Over time, incidence rate decreased from 30.4 to 18.8 cases in men and from 20.1 to 12.9 cases in women. The mortality rate decreased from 17.5 to 14.4 deaths in men and from 14.2 to 10.9 deaths in women. The incidence trend was composed of a first period (1986-1999) of strong decline (EAPC Men= -2.6, 95% Confidence Interval [CI]: -4.2, -0.9; EAPC Women= -3.2, 95% CI: -4.6, -1.9), followed by a less important decrease in men (EAPC= -0.8, 95% CI:-2.5, 0.9) and a slight increase in women (EAPC= 0.7, 95% CI: -1.4; 2.8). Mortality rates were constantly decreasing in both men (EAPC= -0.5, 95% CI: -0.9, -0.1) and women (EAPC= -0.9, 95% CI: -1.7, -0.1) throughout the period of analysis.

**Conclusions:**

The declines in incidence and mortality rates are stagnating. It is important to take measures to further reduce the high burden of GC.

## 1. Introduction

Gastric cancer (GC) represents a major challenge to public health throughout the world. It was responsible for over a million new cases in 2018, and an estimated number of 783,000 deaths (equating to 1 in every 12 deaths) globally, making it the fifth most frequently diagnosed cancer and the third leading cause of cancer death [1]. In Quito, the capital of Ecuador, GC represented the second most frequently diagnosed cancer among men and the fourth among women, during the period 2009 to 2013; it was the leading cause of cancer mortality in both men and women in 2015 [2].

GC incidence and mortality rates have decreased in the last three decades worldwide. These decreases have been explained through better living conditions, changes in the conservation, and availability of food, especially fresh fruits and vegetables, followed by a decrease in the primary etiological factor:* Helicobacter pylori* [3, 4]. Despite this favorable trend, there is still a great geographical variability in the incidence and mortality rates between regions and countries [3, 5] and inside countries [6, 7]. Central and South America (CSA) and Eastern Asia have the highest incidence rates in the world, whereas, North America, Western Africa, and South-Central Asia have the lowest rates [3]. Among CSA countries, incidence varied 6-fold (from Age Standardised Rate (ASR) of 3 in Guyana to 17.8 in Chile) and mortality 5–6-fold (ASR 2.9 in Puerto Rico, to 12.8 in Peru) [1]. Males had up to 3 times higher rates than females [3]. Mortality rates of GC in CSA are the highest along the Pacific coast, with the highest mortality rates (from 12.4 to 22.3 per 100,000) in the Andes Mountains (from Venezuela to Chile) and the Sierra Madre Mountains in Central America (from southern Mexico to Costa Rica) [3, 8].

The main known etiological factors related to the development of gastric cancer are infection by* Helicobacter pylori* and diet. The elevated consumption of salted foods, meat, and refined carbohydrates are directly associated with the risk of developing this neoplasm, while a diet based on fiber, vegetables, and fresh fruit decreases this risk [3, 4]. Usually, gastric cancer is also associated with low socioeconomic conditions [5], an aspect that highlights the importance of analyzing incidence and mortality in countries characterized by high socioeconomic inequalities. Despite reported decreases in overall incidence and mortality rates for gastric cancer [2, 3, 9, 10], recent projections indicate that the burden of stomach cancer in CSA in terms of number of patients will increase by approximately 80% by the year 2030 (102,000 new cases and 88,000 deaths). These increases are expected to be driven primarily by the growth and ageing of the population [3, 4]. Despite the importance of exploring long-term periods of incidence and mortality rates that allow the patterns determination and the identification of changes in trends and discern patterns by calendar period and birth cohort, as essential measures to support public policy actions, no studies have been published exploring the trends of GC within Ecuador until now. In this sense, we present an indepth analysis of time trends, including an Age-Period-Cohort analyses, from 1985 to 2013, to understand the pattern of GC.

## 2. Methods

The Quito Cancer Registry (QCR) records all cases of cancer diagnosed in the city of Quito, using a methodology which is based on international standards, established by the International Association of Cancer Registries (IACR), as part of the International Agency for Research on Cancer (IARC) [11]. The information is obtained through an active process, in which a group of registrars extracts information from the pathology, hematology, and cytology laboratories of all public and private health center establishments in Quito performing on-site active case-finding [2]. The QCR codes its cases using the International Classification of Diseases–Oncology version III (ICD-O3) [11]. The information collected is validated through several quality controls. At the end of each year, the validation tool IARCcrgTools 2.05 is used to establish the coherence of the main variables that should have a population base register [12]. The registry has published its data in the volumes VI to XI of the series of cancer incidence in five continents (CI5), edited by the International Agency for Research on Cancer (IARC) and by the International Association of Cancer Registries IACR, indicating it complies with the quality standards necessary to be included in this publication [10]. Out of all cases registered by the QCR, 87.4% had microscopic verification of a tissue specimen and 6.8% of the cases were based on death certificate only (DCO cases) [13].

### 2.1. Cases

Cases of gastric cancer (C16.0-9) were obtained from the database of the population-based Quito Cancer Registry (QCR). The analysis included all anatomical and histological subtypes of GC with the exception of lymphomas occurring in the stomach. Information on age and sex was available for more than 99.0% of cases.

During the initial years of the cancer registry, the information on sublocalization in gastric cancer was unavailable for 90% of the cases. This value decreased progressively to 59% for the year 2013. As many cases lacked this information, we did not perform trend analysis by anatomical subsite.

### 2.2. Population

The city of Quito is the capital of Ecuador; it is the second largest and most populated city in the country. It is located at latitude 0 (0°13′23′′ South), west of the Andes Mountain Range, at 2,800 meters above sea level. The extension of the city is 127 Km2 and its population for the year 2013, according to the Census Projections of the year 2010, was 1,694,086 inhabitants [14]. The largest percentage of the population of Quito identifies itself as mestiza (80.6%), 12.8% as white, 3.3% as indigenous, and 3.1% as Afro-descendant. Regarding the level of education, 2.7% of the population are illiterate, 30.9% have primary education, 39.7% have secondary education, and 26.7% have higher education [14]. Data on midyear population counts by age and sex were obtained from the National Institute of Statistics and Census (INEC), which contains annual demographic information [14]. The QCR database is linked annually with that of the Civil Registry to determine the vital status of the registered subjects.

### 2.3. Analysis

Incidence and mortality rates, standardized by age (ASR, expressed per 100,000 person-years), were calculated by the direct method, using the Segi world standard population [15]. ASR was also calculated by subtype: intestinal and diffuse type GC. The time trends were evaluated through the estimated annual percentage change (EAPC), using the joinpoint regression program [16]. The EAPC represents the percentage of average increase or decrease in cancer rates per year, over a specific period of time. Significance tests were performed using the Monte Carlo permutation technique. EAPC and joinpoints were determined based on logarithmically transformed ASRs and their standard errors. We specified a maximum of four points of union with at least five observation points.

To conduct Age-Period-Cohort (APC) analysis, the mortality, incidence, and population data of GC were arranged into consecutive 5-year periods from 1985 to 2013 and successive 5-year age intervals from 25-29 years to 70–74 years (individuals over 80 were not considered in this study since they were only recorded as one group in the Population Census database). This method aims to quantify the respective effects of age at diagnosis, calendar year of diagnosis, and birth cohort in the overall population cancer rates. We obtained the estimable parameters from the Age-Period-Cohort Web Tool16, provided by the Division of Cancer Epidemiology & Genetics of the National Cancer Institute [17]. Wald Chi-Square tests were adopted for the significance of the estimable functions. All statistical tests were two-sided, and P < 0.05 was considered statistically significant. Period rate ratio curve describes the relative rate of cancer in any given calendar period versus a referent period (1999 for Incidence and 1998 for Mortality), adjusted for age and nonlinear cohort effects. Cohort rate ratio curve describes the relative rate of cancer in any given birth cohort versus a referent cohort (1951 for incidence, 1950 for mortality), adjusted for age and nonlinear period effects [17].

The data covered the following six periods: 1985-1989, 1990-1994, 1995-1999, 2000-2004, 2005-2009, and 2010-2013 for all GC types. The analysis by histological subtype was carried out from 1995 onwards—prior to this period, histological information was not available in the cancer registry database.

## 3. Results

### 3.1. Incidence and Mortality

A total of 6,795 cases of gastric cancer were diagnosed between 1985 and 2013 (3627 cases among men, 3168 cases among women).  Table 1 shows the distribution of incidence and mortality cases according to gender, age group, and period of time. Over time, the incidence rate decreased from 31.1 to 20.8 cases in men and from 22.3 to 14.0 cases in women. The incidence trend was composed of a first period (1986-1999) of sharp decline (Men EAPC = -2.6, 95% CI -4.2, and -0.9; Women EAPC = -3.2, 95% CI -4.6, -1.9), followed by a nonsignificant decrease in men (EAPC = -0.8, 95% CI -2.5, 0.9) and a slight, nonsignificant increase in women since 2001 (EAPC = 0.7, 95% CI: -1.4, and 2.8).

**Figure 1:**
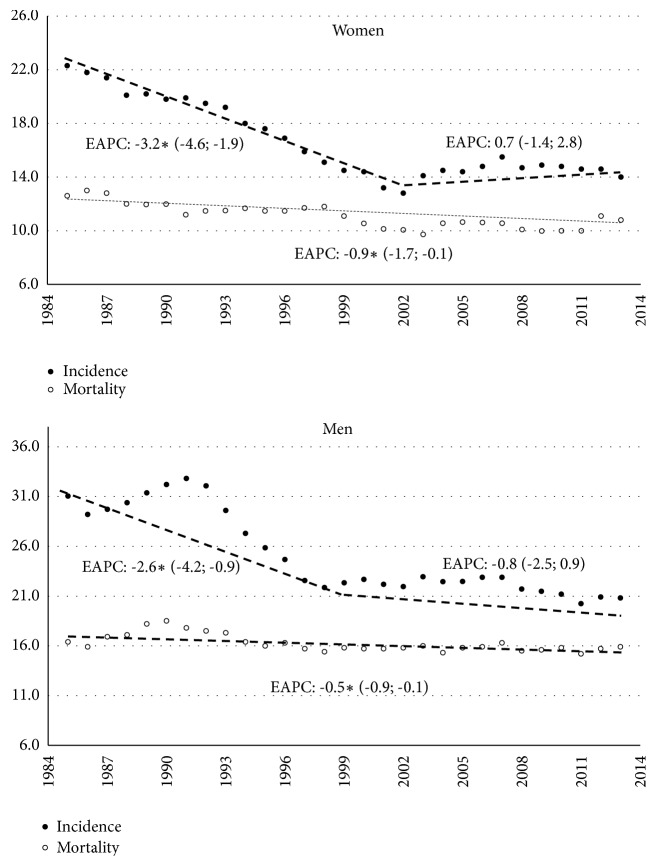
Standardized incidence and mortality rates for gastric cancer in women and men, including EAPC based on joinpoint models. Y: Age-standardized rates per 100,000 person-years; X: Year of diagnosis/death. EAPC: Estimated Annual Percent Change, figures between brackets are 95% CI of EAPC; a star indicates statistical significance at *α* 0.05.

During the same period, a total of 4451 deaths were registered (2493 deaths among men, 2216 deaths among women). The age-standardized mortality rate decreased from 17.5 to 14.4 deaths in men and from 14.2 to 10.9 deaths in women. Between successive periods of 5 years, mortality rates decreased steadily and significantly, both in men (EAPC = -0.5, 95% CI: -0.9, and -0.1) and in women (EAPC = -0.9, 95% CI: -1.7, and -0.1) (Figure 1).

### 3.2. Age-Period-Cohort (APC) Analysis

In both incidence and mortality, there is a remarkable period and cohort effect. In men, the period RR peaked in 1994, subsequently fell until 1999, and stabilized thereafter. In women, the period RR decreased continuously until 2004, then increased, and remained above 1.0 thereafter.

**Figure 2:**
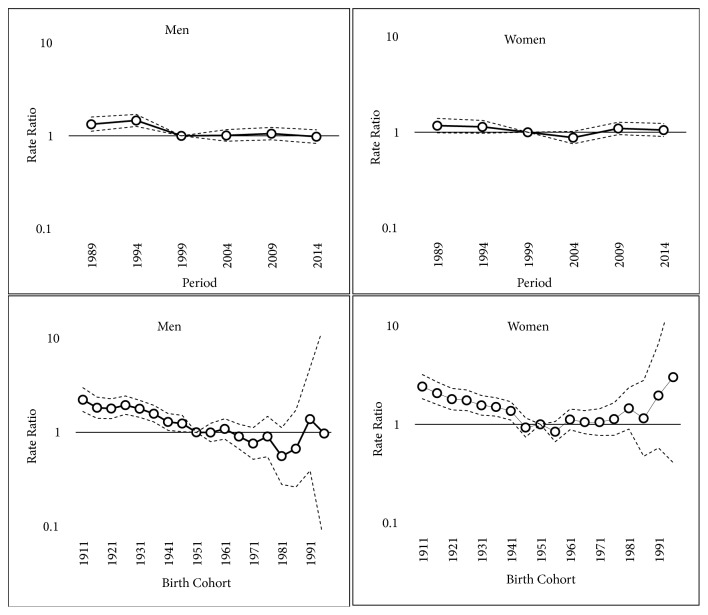
Period and birth cohort effect estimates, with their respective confidence intervals, for the incidence of gastric cancer by sex.

**Figure 3:**
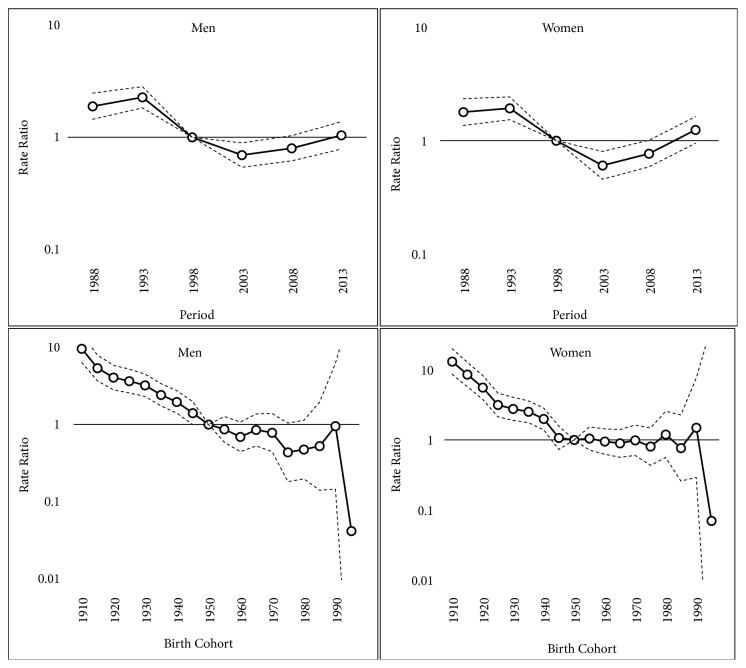
Period and birth cohort effect estimates, with their corresponding confidence intervals, for the mortality of gastric cancer by sex.

There was a significant decrease in the cohort RR, between the 1911 and the 1951 birth cohorts. The cohort effect was stronger in women. Since the beginning of the 20th century, the risk of suffering and dying from gastric cancer decreased in successive generations up to the 1951 cohort, after which the risk stabilized (Figures 2 and 3).

### 3.3. Incidence by Subtype

**Figure 4:**
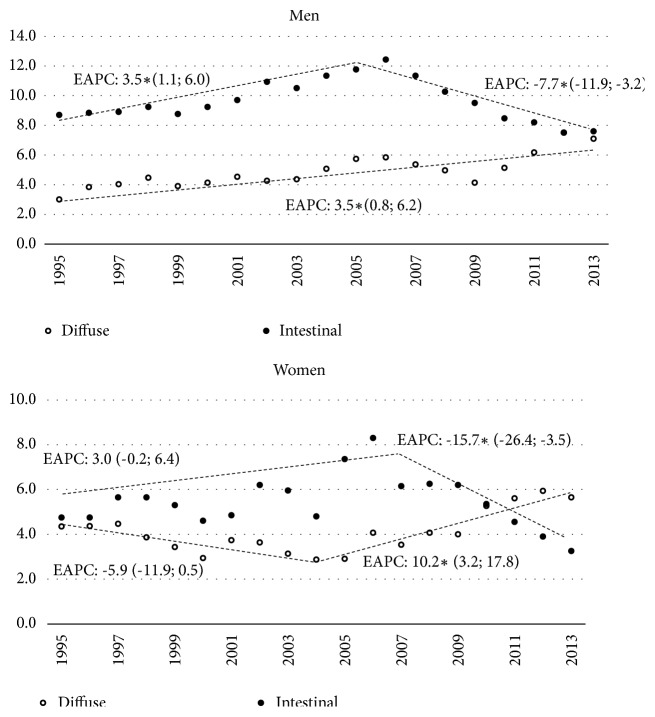
Standardized incidence rate trend for gastric cancer in women and men, by subtype. Includes EAPC based on joinpoint models. EAPC: Estimated Annual Percent Change, figures between brackets are 95% CI of EAPC; a star indicates statistical significance at *α* 0.05.

Table 2 shows the standardized incidence rates per 100,000 inhabitants, according to sex and year, of the intestinal and diffuse types of the gastric cancers. Temporal trends in incidence varied according to the subtypes. As shown in Figure 4, the intestinal type has a first period (1995-2005) of significant increase in men (EAPC: 3.5, 95% CI 1.1, 6.0) and a nonsignificative increase (EAPC: 3.0 95% IC -0.2, 6.4) in women (1995 to 2007), followed by a sharp decline in both sexes.

The incidence rates of diffuse type increase constantly in men throughout the whole period; in women, the increase has been more marked since 2004 (Figure 4).

## 4. Discussion

During a period of 29 years, the incidence and mortality of gastric cancer decreased in Quito–completely in line with global observations of declining trends [3, 10]. However, the most outstanding and worrying aspect of these results is the stagnation in the decline of the trends observed in recent years. The stagnation becomes evident around the year 2000, in both men and women. Considering the continuous improvements in the living conditions of the Quito population [14, 18], a continuous decline in incidence and mortality was expected; however, the observed stagnation indicates that action is warranted to establish a continuous decline.

During the 80s, Ecuador underwent a process of structural adjustment to overcome the economic crisis [19]. In addition to the social conflicts that occurred and an environment of political instability in the 1990s, the economy and the state implemented neoliberal measures [20]. In short, the set of economic, state, and social policies forged in the golden decades of neoliberalism laid the foundations to sustain the transformation of human and social rights into commodities [20, 21]. At the end of the 1990s, the Ecuadorian situation worsened due to several factors, including the El Niño phenomenon of 1998 and the fall in oil prices in 1998-1999. Between 1999 and 2000 the financial system was affected by the closure of more than half of the country's banks, suspending banking operations [19]. In the year 2000 the currency was officially dollarized when a dollar was equivalent to 25,000 “sucres.” This situation has had a high social cost, in terms of an increase in inequality and the persistence of poverty and unemployment thereafter [19–21].

The available data on the risk factors for GC allowed us to interpret the trends by birth cohort and diagnostic period. Cohort and period effects have different causes and implications. For example, cohort effects are usually related to exposure to early-stage risk factors or protective factors, with a long-term latency period that affects each birth cohort (generation) slightly differently. Conversely, a period effect can be attributed to risk factors or protective factors involved in later stages of carcinogenesis that affects all age groups, or to changes in detection and diagnosis practices that were applied cross-sectionally to the entire population [22].

We observed a significant decrease in the cohort RR between those born in 1911 and those born in 1951. In cohorts born after 1951, the continuous decline in both incidence and mortality stagnates. This situation is similar to what happened in some European countries, but with a delay of a few decades and remaining rates of gastric cancer being much higher in Quito. For example, data from the Zaragoza cancer registry (Spain) showed an increase in risk of GC in people born after 1930, compared to previous generations [23]. A study of the secular trend of gastric cancer in six European countries showed that rates in people born after 1930 increased in Scotland and France, but also, although less markedly, in Germany, the Netherlands, Spain, and Sweden [24].

It is well established that, on a global scale,* Helicobacter pylori* infection is the main risk factor for gastric cancer [25]. Infection with* Helicobacter pylori* leads to a long carcinogenic process that starts with chronic gastritis, which can progress to atrophic gastritis, intestinal metaplasia, dysplasia, and gastric cancer [26]. The decrease of* Helicobacter pylori* infection in childhood, for successive birth cohorts, is considered as the main explanation of the observed cohort effects globally [27]. This decrease is mainly the result of better living conditions, with better hygiene and sanitation. The decrease in salt consumption throughout the generations can also contribute to the cohort effect for GC [28].

The prevalence of* Helicobacter pylori* infection varies widely among countries and within countries by age, race or ethnic group, migration from areas of high prevalence and indicators of low socioeconomic status (agglomeration, level of education, lack of adequate sanitation and drinking water) [29, 30]. In South America, for example, the prevalence of* Helicobacter pylori* infection among adults ranges between 50% and 95% [31]. In Ecuador, a study developed in 2004, showed a* Helicobacter pylori* seroprevalence of 63% in children less than 16 years at the national level [32]. Sasaki et al. reported a* Helicobacter pylori* infection rate of 72.2% by the year 2009. In the same study, among* Helicobacter pylori* DNA-positive samples,* cagA* was detected in 45.9%. Over 80% of the detected* cagA *was East-Asian genotype, which has been considered of high virulence [33, 34].

The urban expansion of Quito, during the 20th century, resulted in a continuous absorption of the indigenous populations of the periphery of Quito [35]. The population of Quito grew from 275399, in 1950, to 2239191 people in the year 2010 [36]. According to the 2010 population census, 69.8% of the population of Quito completed basic education, which is more than the national average of 54.0%. While at the national level poverty, measured through Unsatisfied Basic Needs (NBI), reached 60.1% of the population, in the city of Quito this indicator barely reached 29.7%. The city has the largest coverage of potable water and sewage services in the national context [18, 37]. Improvements in living conditions in Quito may have played an important role in the gradual decrease in the incidence of gastric cancer, if they had been equally distributed in the population.

In addition, the improvement of public education in different birth cohorts should also be considered in the decrease of cohort RR. Since the 1950s, Ecuador, like most other Latin American countries at that time, adopted development planning measures, which included an extension of public education, conceived as a creator of wealth and social stability [38]. In the field of education, the theoretical substrate of the new development paradigm is the theory of human capital, through which attempts were made to match educational reforms with the requirements of the occupational system; these decisions were understood as capital investments [38]. From there, the new more economistic and practical conception of education prompted important reforms that implied a change in the distribution of the population, according to their levels of study as population heterogeneity increased. Relevant information indicated that education in the birth cohort 1950s and 1960s had been greatly improved compared with those cohorts born before 1949, which benefited from the vigorous promotion of literacy and the positive development of basic education [38, 39]. A higher level of education is generally regarded as a protective factor for GC, but due to a lack of data, we were unable to directly examine this hypothesis. Although we cannot be certain, it seems likely that the continuous improvement of public education may have contributed to the decreasing trends in GC in Quito as evidenced by the cohort effect change seen here.

Regarding GC subtypes, we found a significant increase of incidence rates for diffuse type, in both men and women. In general, the diffuse type is diagnosed in young adults due to its relationship with the hereditary background. In the United States, the Epidemiology Surveillance and Final Results (SEER) population registries of the National Cancer Institute showed that the incidence rates of gastric cancer in people older than 40 years continued to decrease between 1977 and 2002. But the rate increased in that period in people between 25 and 40 years of age [40]. A similar situation was reported also in Colombia [41]. In Central and South America, the intestinal subtype was 2-7 times more common than the diffuse type in the last decade [3]. Our recent observations show that this relationship is beginning to reverse, being stronger in women than in men.

Among Latin American countries, there has been an evident increase in mortality rates among rural populations with a lower level of education [42–45]. The highest mortality rates observed in lower socioeconomic groups (SES) are likely due to a combination of higher incidence rates and worse survival expectation [46]. In this context, the deceleration of the decrease in current trends reflects the need to deepen the analysis in terms of social inequalities, in order to understand the social logic that underlies the inequalities, as well as to focus and strengthen primary and secondary prevention strategies.

Until recently, the most well-known strategies to prevent GC, apart from reducing the prevalence of chronic* Helicobacter pylori* infection, included limiting consumption of canned and salted foods, eating more fruits and vegetables, and not smoking. However, a more active approach to the treatment of* Helicobacter pylori* for the prevention of stomach cancer is currently being evaluated. In recent randomized trials, it has been shown that the screening and eradication of* Helicobacter pylori* with antibiotics reduce the risk of stomach cancer [47]. Although this approach requires more research, it may represent a promising new way to further reduce the rates of stomach cancer in countries where chronic* Helicobacter pylori* infection is common.

We need to keep in mind the change in the completeness of cancer registration in QCR when we evaluate incidence data. Through the study period, the percentage of microscopic verification of a tissue specimen increased from 70.4% to 82.6% in men and from 57.2% to 81.3 in women; the percentage of DCO decreased from 19.5 to 11.1 in men and from 30.7 to 11.9 in women [13]. A limitation to be considered in the study is the impossibility of separating cardia and non-cardia locations for gastric cancer, which seem to present different behaviors according to most recent studies [3]. Another important fact to consider is the lack of historical series for risk factor prevalence within Ecuador, which could aid in the analysis of the observed changes.

The RCQ is the oldest population-based cancer registry in Ecuador. The multiplicity of sources of information allows us to suppose that almost all newly diagnosed gastric cancers were registered. The strength of our research lies in the fact that the registration scheme and coding rules remained the same during the study period. Moreover, this is the first published study, to our knowledge, to investigate the long-term incidence and mortality trends of gender-specific GC in Latin America and to examine the Age-Period-Cohort effects of them under the APC framework.

In conclusion, the previously observed reductions in mortality and incidence did not continue. It is important to take measures to reduce the high burden of this lethal disease in the population of Quito and probably in many cities and countries in similar situations.

*Human Rights*. This article is based on secondary analysis of data on cases/deaths and population counts in aggregate form made publicly available by the Quito Cancer Registry and National Institute of Statistics and Census, respectively. All procedures followed were in accordance with the ethical standards of the responsible committee on human experimentation (institutional and national) and in compliance with the Helsinki Declaration of 1964 and later versions.

## Figures and Tables

**Table 1:** Gastric cancer cases (incidence cases) and deaths (mortality cases) by sex, age group, and period of time, Quito, 1985–2013.

Sex	Age group	Period											
		1985-1989		1990-1994		1995-1999		2000-2004		2005-2009		2010-2013	
		Inc	Mor	Inc	Mor	Inc	Mor	Inc	Mor	Inc	Mor	Inc	Mor
Men	< 30	6	2	10	4	7	1	14	10	10	5	13	6
	30-34	17	7	16	10	10	6	10	8	14	10	11	8
	35-39	10	6	21	10	13	9	16	10	15	12	11	7
	40-44	16	10	23	10	23	13	39	25	30	15	23	13
	45-49	23	13	25	13	32	19	29	21	51	32	40	26
	50-54	43	25	36	20	44	24	46	32	56	39	45	26
	55-59	47	33	53	21	45	34	64	40	71	47	54	41
	60-64	55	40	78	48	70	47	54	41	75	51	53	33
	65-69	60	35	79	43	65	53	62	43	91	53	90	59
	70-74	58	34	78	50	75	60	91	69	109	75	89	62
	75-79	64	44	68	42	55	45	102	89	109	80	80	56
	> 80	67	51	86	65	95	78	134	116	140	124	113	84
	Total	466	300	573	336	534	389	661	504	771	543	622	421

Women	< 30	12	5	12	6	7	4	9	7	8	6	16	8
	30-34	5	2	15	4	16	9	12	9	23	17	25	14
	35-39	20	10	12	8	28	17	22	15	32	22	24	13
	40-44	17	11	24	8	23	16	33	23	30	15	32	21
	45-49	21	13	21	10	28	17	21	16	39	25	46	30
	50-54	34	18	32	18	26	19	32	23	40	26	42	27
	55-59	20	11	38	23	48	31	29	24	45	29	47	27
	60-64	39	25	50	30	52	38	45	29	56	30	44	27
	65-69	54	37	56	37	55	46	40	32	84	55	43	19
	70-74	59	36	54	32	51	36	63	50	86	54	62	43
	75-79	69	54	58	45	60	48	67	55	75	56	48	34
	> 80	86	68	96	70	114	104	138	120	158	135	140	114
	Total	436	290	468	291	508	385	511	403	676	470	569	377

Inc: incidence cases and Mor: mortality cases.

**Table 2:** Standardized incidence rates for gastric cancer subtype in women and men, 1995–2013.

	Intestinal		Diffuse	
year	Women	Men	Women	Men
1995	3.8	8.6	4.2	3.9
1996	5.7	8.8	4.5	2.1
1997	5.6	9.1	4.4	5.5
1998	5.7	8.8	4.5	4.5
1999	4.9	9.8	2.7	3.4
2000	4.3	7.7	3.1	3.8
2001	5.4	10.2	3.0	5.2
2002	7.0	11.2	5.1	4.6
2003	4.9	11.4	2.8	3.0
2004	4.7	8.9	1.5	5.5
2005	10	13.7	4.3	6.7
2006	6.6	12.7	2.9	5.0
2007	5.7	10.9	5.0	5.8
2008	6.8	10.4	2.7	5.3
2009	5.6	9.5	4.5	3.8
2010	5.1	8.6	4.8	3.3
2011	4.0	7.3	6.5	8.3
2012	3.8	8.7	5.5	6.9
2013	2.7	6.5	5.8	7.3

## Data Availability

The crude data of cancer cases and deaths used to support the findings of this study may be released upon application to the “Registro Nacional de Tumores”, who can be contacted through the corresponding author, or at the following e-mail: rnt@solcaquito.org.ec.
